# Targeted quantitative mass spectrometric immunoassay for human protein variants

**DOI:** 10.1186/1477-5956-9-19

**Published:** 2011-04-08

**Authors:** Olgica Trenchevska, Dobrin Nedelkov

**Affiliations:** 1 Intrinsic Bioprobes, 2155 E. Conference Dr. Suite 104, Tempe, AZ 85284, USA

## Abstract

**Background:**

Post-translational modifications and genetic variations give rise to protein variants that significantly increase the complexity of the human proteome. Modified proteins also play an important role in biological processes. While sandwich immunoassays are routinely used to determine protein concentrations, they are oblivious to protein variants that may serve as biomarkers with better sensitivity and specificity than their wild-type proteins. Mass spectrometry, coupled to immunoaffinity separations, can provide an efficient mean for simultaneous detection and quantification of protein variants.

**Results:**

Presented here is a mass spectrometric immunoassay method for targeted quantitative proteomics analysis of protein modifications. Cystatin C, a cysteine proteinase inhibitor and a potential marker for several pathological processes, was used as a target analyte. An internal reference standard was incorporated into the assay, serving as a normalization point for cystatin C quantification. The precision, linearity, and recovery characteristics of the assay were established. The new assay was also benchmarked against existing cystatin C ELISA. In application, the assay was utilized to determine the individual concentration of several cystatin C variants across a cohort of samples, demonstrating the ability to fully quantify individual forms of post-translationally modified proteins.

**Conclusions:**

The mass spectrometric immunoassays can find use in quantifying specific protein modifications, either as a part of a specific protein biomarker discovery/rediscovery effort to delineate the role of these variants in the onset of the disease, progression, and response to therapy, or in a more systematic study to delineate and understand human protein diversity.

## Background

Protein modifications play important roles in biological process, and can serve as diagnostic indicators of pathological events [1]. However, their detection and quantification is not straightforward because oftentimes these modified forms are only slightly chemically different from their wild-type proteins. Powerful separation methods are thus needed to differentiate protein variants in preparation for subsequent detection. Alternatively, mass spectrometry can detect all protein variants in a single analysis; a MALDI mass spectrum can contain signals from multiple protein variants, as long as the variants (along with the targeted native protein) are not hindered by other overabundant proteins which can dynamically suppress their signals. In that case, some sort of fractionation is still needed, especially when analyzing complex samples such as human plasma and serum. Immunoaffinity separation offers simple and targeted isolation of proteins and their variants in preparation for MS detection, by using antibodies toward invariable epitopes in the protein sequence. The result of one such combination of immunoaffinity separation and mass spectrometric detection is the Mass Spectrometric Immunoassay [2,3], which can offer something that conventional enzymatic immunoassay cannot - detection and quantification of protein variants. The secondary antibody used in sandwich immunoassays cannot discriminate between structural protein modifications and the resulting quantitative signal is the sum of signals from all variants for a given protein captured by the primary antibody. On the other hand, the mass spectrometric analysis can detect all protein variants affinity-retrieved by the capturing antibody. Presence of signals in the mass spectra that do not correspond to the one empirically calculated from the native protein sequence is the first indication of protein variants existence. These signals can be initially assigned to specific variants and modifications by accurate measurements of the observed mass shifts and knowledge of the protein sequence and possible modifications, and are further verified using proteolytic digestion and mapping experiments [4-6].

Presented here is a quantitative mass spectrometric immunoassay for cystatin C variants. Cystatin C is a serine proteinase inhibitor belonging to the type 2 cystatin gene family [7,8]. It is a non-glycosilated single chain protein with a molecular weight of 13,343. Cystatin C has been indicated in numerous pathological states [9-11], most notably in renal failure [12,13]. A variant of human cystatin C (L68Q) is an amyloidogenic protein deposited in the cerebral vasculature of patients with hereditary cerebral hemorrhage with amyloidosis [14-16]. We have also reported on the existence of several cystatin variants [17,18], using a qualitative cystatin C assay. In this work we present the development, full characterization, and validation of quantitative mass spectrometric immunoassay for cystatin C and its variants, and apply the assay to determine the individual human plasma concentration of several cystatin C variants.

## Results

### Internal reference standard

In the past we have utilized homologous proteins from other animal species that are recognized by the same antibody that binds the human antigen, so that upon spiking of the IRS into the analytical sample, the human protein and its animal homologue are retrieved by the single antibody. The two proteins register in the same region of the mass spectrum, but at a slightly different m/z value [4,19,20]. However, this approach has its shortcomings in that the concentration of the IRS can oftentimes vary in different lots of animal sera (if purified protein is not available, as often it is the case). Furthermore, each protein assay requires different IRS, which by itself complicates the development of multiple assays. Hence, we took on the novel idea that a single, unrelated protein may serve as an IRS if an antibody toward that protein is co-immobilized with the antibody toward the targeted human protein, and the analytical samples are spiked with constant amounts of that IRS. An important prerequisite for this type of IRS is that it should not be present in human plasma or serum, so that its spiked concentration in the analytical samples is always constant. Also, the IRS should produce signals in the mass spectra that are in close proximity to the signal of the targeted protein, so that the same MS acquisition parameters can be used for both proteins. We selected beta-lactoglobulin as an IRS for the cystatin C assay (and potentially other assays as well). Beta-lactoglobulin (BL) is the major whey protein of cow's milk, and is also present in many other mammalian species, but not in humans. It is easily obtainable in large quantities and is very affordable (another important factor in the design of the assay). It has a molecular weight of 18,281, which is relatively close to that of human cystatin C (MW = 13,343).

### Assay optimization

The first step in the assay optimization was determination of the ratios of the immobilized cysC and BL antibodies in the affinity pipettes. The general idea is that there should be more CysC antibody so that it can accurately quantify the varying concentrations of CysC in the samples; and less BL antibody because the amount of BL spiked in the samples can be easily controlled. After several empirical iterations, which involved immobilizing both CysC and BL antibodies in various mass ratios onto the microcolumns, it was determined that the optimal mass ratio of the two antibodies was 8.5:1 (CysC:BL). The optimal concentration and volume of BL spiked into the samples were determined next, by starting first with fixed spike volumes containing increasing BL concentrations (i.e., 1 mg/L, 10 mg/L BL, etc), and then using fixed concentrations of BL but increasing the spiking volumes (5, 10 μL, etc.). From these experiments, the optimal BL concentration and volume were determined to be 10 mg/L and 5 μL, respectively. At this concentration, the BL present in the analytical sample saturates the anti-BL antibody, thus producing constant signals in the resulting mass spectra (this is an important prerequisite for the IRS). Next, various volumes (e.g., 5, 10, 20 μL) and dilutions (undiluted, 5-times and 10-times diluted) of human serum or plasma were utilized to determine the optimal sample volume into the analytical samples. It is very important not to saturate the anti-CysC antibody so that increased concentrations of cysC can be readily quantified with the assay. It was determined that 5 μL of serum or plasma, diluted 2-fold into assay buffer, provided the best results. At normal concentrations of cystatin C in plasma (~1 mg/L), these volume and concentration translate into less than 200 fmoles of cystatin C in the analytical samples.

### Standard curve

**Figure 1:**
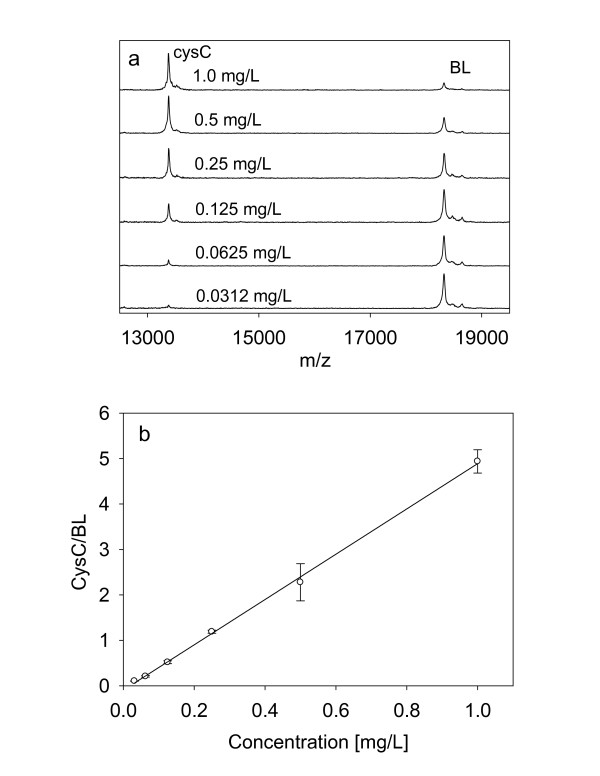
**(a) Representative cystatin C standards mass spectra, and (b) Standard curve generated with the cystatin C (cysC) mass spectrometric immunoassay by using beta-lactoglobulin (BL) as an internal reference standard**.

The standard curve was optimized and determined in parallel with the sample volume optimization. After several iterations, a 6-point standard curve was selected, spanning the range from 0.0312 to 1.0 mg/L cystatin C. An example of the standard curve, along with representative mass spectra, is shown in Figure 1. The response is linear across the entire range, with a coefficient of determination (R^2 ^= 0.998) and standard error of estimate (SEE = 0.0708). It was found that that this range of the standard curve was sufficient to determine the concentration of cystatin C in all of the examined human plasma and serum samples. From the ratios of the CysC/BL peak heights and using a standard curve that was generated in parallel with the analytical samples analyses, the concentrations of cystatin C and its variants in the samples were determined. Operationally, we first determined the CysC/BL peak heights ratios for each cysC form in the sample, summed them up, determined the total cystatin C concentration using the standard curve, and then determined the concentration of the individual forms based on their percentage of the total cystatin C concentration.

### Cystatin C isoforms detection

**Figure 2:**
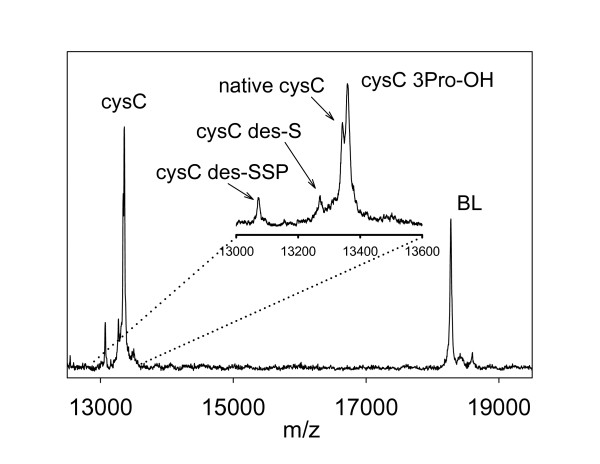
**Typical mass spectrum resulting from analysis of cystatin C from a human plasma sample**.

A typical mass spectrum resulting from the cystatin C mass spectrometric immunoassay of a human plasma sample is shown in Figure 2. Clearly present are signals for BL and CysC along with several cystatin C variants: CysC containing hydroxyproline at position 3 (CysC 3Pro-OH; MW = 13,359), CysC missing its N-terminal Serine (CysC des-S, MW = 13,260), and CysC missing its three N-terminal residues (CysC des-SSP, MW = 13,076). These variants have been detected and identified in previous studies [17,18]. Because CysC 3Pro-OH exhibits similar mass to that of the L68Q variant (MW = 13,358), we performed tryptic digests and peptide mapping experiments to confirm the identity of the hydroxyl-CysC in each sample (results not shown).

### Assay parameters

**Figure 3:**
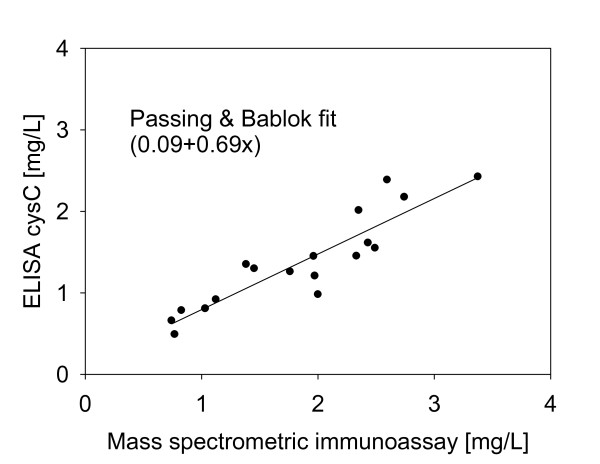
**Cystatin C mass spectrometric immunoassay and standard ELISA method comparison**.

The intra-assay precision (within-run) was determined by analyzing three plasma samples, in triplicates, each with a single standard curve. The inter-assay precision (run-to-run) was determined by analyzing one plasma sample three times, on different days, with separate standard curves each time. The results are shown in Table 1, and indicate CVs of less than 10%. To determine the linearity of the assay, serum samples with known cystatin C concentration were serially diluted, analyzed with the mass spectrometric immunoassay to determine the cystatin C concentrations, and the results compared to those expected (Table 2). Spiking recovery experiments were also performed by spiking serum samples with different amounts of recombinant human Cystatin C, followed by analysis with the assay to determine the total cystatin C concentration, and comparison of the results with those expected (Table 3). In a final test of the assay, eighteen serum samples were analyzed both by the mass spectrometric immunoassay and a commercially available ELISA, and the cystatin C concentrations determined with the two methods were compared. The graph shown in Figure 3 indicates good correlation between the two set of numbers (Passing & Bablok fit of 0.09+0.69x) [21], validating the results obtained with the new cystatin C assay.

**Table 1:** Intra-and inter-assay precision.

**Intra-assay CVs**				**Inter-assay CV**	
	
Sample	1	2	3	STDV	0.040
				MEAN	0.380
STDV:	0.002	0.012	0.005	CV	9.84
MEAN:	0.414	0.327	0.413		
CV:	0.497	3.82	1.14		

**Table 2:** Assay linearity.

Sample	Dilution	Observed	Expected	Recovery
		mg/L	mg/L	O/E %
1		0.709		
	2x	0.316	0.355	89.0
	4x	0.168	0.177	94.9
	8x	0.093	0.089	104

2		0.514		
	2x	0.273	0.257	106
	4x	0.122	0.129	94.6
	8x	0.071	0.064	110

**Table 3:** Spiking recovery.

Sample	Observed	Expected	Recovery
	mg/L	mg/L	O/E%
1	0.326		
	0.704	0.826	85.2
	1.41	1.33	106
	1.72	1.83	94.2

2	0.530		
	0.736	0.780	94.4
	1.02	1.03	99.0
	1.41	1.53	92.2

### Human plasma samples screening

The fully-developed, characterized, and validated cystatin C mass spectrometric immunoassay was then used to analyze a set of human plasma samples in a high throughput mode. Forty-four sodium heparin plasma samples were utilized to prepare 88 analytical samples (each sample was prepared in duplicate, at different dilutions) and placed in columns 2-12 of a 96-well microplate. The standard curve samples were placed in column 1, along with a control sample with a known cystatin C concentration. The 96 cystatin C assays were executed in parallel using the Multimek 96 channel pipettor. Following mass spectrometry analysis and spectra processing, a standard curve was constructed from the data in column 1, and the performance of the assay verified with the control sample. The concentrations of cystatin C and its variants were then individually determined, averaged (from the two analyses for each sample) and are presented in Table 4. The average of the total concentration of cystatin C in the samples is 0.94 mg/L, and correlates well with that previously established (~0.85 mg/L) using ELISA approaches [22]. The major fraction of cystatin C is cysC 3Pro-OH, followed by the native form. The native CysC to CysC 3Pro-OH ratio is ~1:1.25, which agrees with previously reported values for the two [23,24]. The two truncated forms (des-S and des-SSP) are present in all samples, albeit at much lower concentration (each is less than <10% of the total CysC). One sample (#44) also contains point mutations that results in a -30Da peak shift (mass spectra not shown). This mutation was detected in our previous study [18], and again, it was observed only in a sample obtained from a male subject. In all, the assay performed well and was executed in a high-throughput manner: the 96 samples incubation and deposition onto the MALDI target was executed in 30 min, while the mass spectra acquisition/data processing was performed in 3 hours.

**Table 4:** Cystatin C variants concentrations

Sample	native cysC	cysC 3Pro-OH	cysC des-S	cysC des-SSP	Total cysC
	[mg/L]	[mg/L]	[mg/L]	[mg/L]	[mg/L]
1	0.25	0.33	0.07	0.07	0.71
2	0.32	0.41	0.08	0.03	0.84
3	0.39	0.48	0.09	0.08	1.04
4	0.42	0.54	0.08	0.08	1.11
5	0.35	0.46	0.11	0.11	1.02
6	0.27	0.35	0.07	0.09	0.79
7	0.24	0.34	0.06	0.04	0.68
8	0.69	0.83	0.15	0.18	1.85
9	0.45	0.58	0.12	0.11	1.27
10	0.29	0.38	0.08	0.09	0.85
11	0.40	0.55	0.13	0.14	1.22
12	0.40	0.46	0.09	0.09	1.04
13	0.30	0.38	0.07	0.08	0.83
14	0.22	0.30	0.07	0.08	0.67
15	0.36	0.45	0.09	0.10	1.00
16	0.20	0.26	0.09	0.04	0.58
17	0.23	0.31	0.05	0.05	0.64
18	0.38	0.47	0.09	0.09	1.03
19	0.40	0.52	0.09	0.09	1.09
20	0.41	0.53	0.09	0.08	1.11
21	0.38	0.47	0.10	0.07	1.02
22	0.31	0.41	0.08	0.06	0.85
23	0.36	0.50	0.08	0.06	1.00
24	0.34	0.45	0.08	0.09	0.96
25	0.13	0.18	0.04	0.03	0.38
26	0.34	0.43	0.11	0.10	0.98
27	0.28	0.34	0.08	0.07	0.77
28	0.32	0.41	0.09	0.09	0.91
29	0.20	0.25	0.05	0.05	0.54
30	0.33	0.40	0.08	0.07	0.87
31	0.41	0.49	0.08	0.08	1.06
32	0.39	0.52	0.10	0.07	1.09
33	0.32	0.40	0.07	0.07	0.86
34	0.23	0.29	0.09	0.09	0.70
35	0.18	0.22	0.05	0.05	0.50
36	0.48	0.69	0.15	0.12	1.44
37	0.46	0.62	0.12	0.11	1.31
38	0.42	0.56	0.12	0.09	1.18
39	0.23	0.30	0.08	0.09	0.70
40	0.39	0.51	0.09	0.07	1.06
41	0.36	0.43	0.05	0.11	0.95
42	0.24	0.33	0.05	0.04	0.67
43	0.47	0.57	0.11	0.10	1.25
44	0.45	0.49	0.06	0.11	1.12

## Conclusions

The assay presented in this works utilizes mass spectrometry to fully quantify cystatin C variants. Similar MS assays can also be designed for other proteins to enable full quantification of their post-translationally modified forms. There are other affinity-based MS methods for protein quantification. Among the earliest is SELDI, which utilizes MALDI target-bound antibodies for protein retrieval [25]. In a similar approach called iMALDI, affinity beads with immobilized anti-peptide antibodies are utilized to capture targeted peptides and stable-isotopically labeled standards, which are then MS-analyzed when the affinity beads are placed on a MALDI target [26]. Most popular today are approaches based on single/multiple reaction monitoring (SRM/MRM) LC-MS/MS. Even though they can be executed without affinity enrichment (for quantification of medium-to-high level plasma proteins) [27], the immunoaffinity separation is the key enabling step for quantification of low-level plasma proteins. SISCAPA is the most advanced MRM LC-MS/MS approach [28,29]. SISCAPA utilizes isotopically labeled peptides as internal reference standards for surrogate protein quantification via enzymatically-generated peptides which are retrieved by a single antibody. While SRM/MRM LC-MS may be suitable for quantification of low abundance proteins for which ELISAs are not available, they typically quantify the proteins via few surrogate peptide fragments. Hence, a large portion of the protein sequence is excluded from the interrogation, leaving out important information about possible variants with potential clinical implications. Furthermore, their complex, multi-step protein identification process introduces an array of possible paths that can lead to reproducibility issues [30-32]. Finally, the SRM/MRM LC-MS assays are designed *a-priori *for detection of specific peptides, which eliminates the ability to detect novel truncations and mutations. On the other hand, the assay and methodology presented here utilize a simplistic two-step approach to native and intact protein analysis - in many ways similar to that in well established enzymatic immunoassays. However, it is the last step of the assay - the mass spectrometric detection - that enables differentiation of the multiple protein variants. Enzymatic immunoassay are oblivious to protein modifications, unless an antibody is designed that specifically recognizes a single protein variant (a Herculean task by itself). The mass spectrometric immunoassays, on the other hand, are designed with detection of protein modifications in mind. With the added quantitative feature, these assays are poised to change the way we look at protein modifications and their role in diseases - one protein at a time.

## Methods

### Reagents

Rabbit anti-human polyclonal antibody to cystatin C (cysC) was obtained from DAKO (Carpinteria, CA, USA, Catalog No. A0451, 17 g/L). Rabbit anti-human polyclonal antibody to beta-lactoglobulin (BL) was obtained from GeneTex (Irvine, CA, Cat. No. GTX77272, 1 mg/mL). Recombinant human cystatin C was purchased from HyTest (Turku, Finland, Cat. No. 8CY5). Beta-lactoglobulin from bovine milk (Cat. No. L8005), 1,1' Carbonyldiimidazole (115533), TWEEN 20 (P7949), TRIS (T-6128), Dithiothreitol (DTT, 43815) and α-cyano -4-hydroxycinnamic acid (476870) were obtained from Sigma-Aldrich (St. Lous, MO). Affinity pipettes fitted with porous microcolumns were obtained from Intrinsic Bioprobes (Tempe, AZ, Cat No. IBI-CMD-R96). Phosphate buffered saline was obtained from Thermo Scientific (Rockford, IL, Cat. No. 28374). Sterile water (Cat. No. AB02120), acetone (AB00636), MES (AB01235), acetonitrile (AB00120) and trifluoracetic acid (AB02010) were purchased from American Bionalytical (Natick, MA). Methyl-1 pyrrolidone-2 was obtained from EMD Chemicals (Gibbstown, NJ, Cat. No. MX1932-5). N-octylglucoside was obtained from Roche Applied Science (Indianapolis, IN, Cat. No. 10634425001). Cystatin ELISA kit was obtained from Biovendor (Candler, NC, Cat. No. RD191009100). Sequencing grade modified trypsin was obtained from Promega (Madison, WI, Cat. No. V511).

### Instrumentation

Derivatization of the affinity pipettes with antibodies and high-throughput mass spectrometric immunoassays were performed on a Multimek 96 automated 96-channel pipettor (Beckman Coulter, Brea, CA). Manual mass spectrometric immunoassays were performed with the help of an 8-channel Finnpipette Novus multichannel pipetter (Thermo Fisher Scientific, Waltham, MA). Mass spectrometry was performed on an *Autoflex *II MALDI-TOF (linear spectra) and *Autoflex *II *TOF/TOF *MALDI-TOF (reflectron spectra) mass spectrometers (Bruker, Billerica, MA). ELISA readouts were obtained on Cary 50 spectrophotometer equiped with a microplate reader accesory (Varian Instruments, Walnut Creek, CA). The cystatin C ELISA was carried out according to the kit manufacturer instructions (Biovendor, Cat. No. RD191009100)

### Human serum and plasma samples

Forty-four Na-heparin human plasma samples and eighteen human serum samples were obtained from ProMedDX (Norton, MA, USA). The samples were collected at certified blood donor and medical centers and designated as normal based on their non-reactivity for common blood infectious agents and the donor information itself. The samples were labeled only with a barcode and supplied with an accompanying specification sheet containing information only about the gender and age, ensuring proper privacy protection.

### Preparation of affinity pipettes

Ninety-six affinity pipettes were mounted on the head of the Multimek 96 pipettor and initially rinsed with 200 mM HCl (20 cycles, each cycle consisting of an aspiration and dispense of a 150 μL volume), followed by a water rinse (5 cycles) and acetone rinse (5 cycles). To activate the surface of the microcolumns contained within, the pipettes were immersed into a tray containing 100 mg/mL 1,1' Carbonyldiimidazole (in methyl-1 pyrrolidone-2), and 500 cycles of 100 μL aspirations and dispenses through each affinity pipette were performed. Two rinses with methyl-1 pyrrolidone-2 (10 cycles each, 150 μL volumes) and a final rinse with acetone (10 cycles, 150 μL) followed. The affinity pipettes were then immediately immersed into a microwell plate containing the antibodies solutions (0.085 mg/mL cystatin C antibody, and 0.01 mg/mL beta-lactoglobulin antibody, in 10 mM MES) and 800 cycles of aspirations and dispenses of 50 μL volumes were performed to bind the antibodies to the activated microcolumns surfaces. Two rinses with 60 mM HCl followed (30 cycles each, 100 μL), ending with two final rinses with assay buffer (PBS w/0.1% TWEEN, 30 cycles each, 100 μL). The antibody-derivatized pipettes were stored at 4°C until used.

### Preparation of standards and analytical samples

For the generation of the standard curve, a solution containing 1.0 mg/L recombinant cystatin C was prepared (Standard #1) and serially diluted with assay buffer to 0.5 mg/L (Standard #2), 0.25 mg/L (Standard #3), 0.125 mg/L (Standard #4), 0.0625 mg/L (Standard #5), and 0.0312 mg/L (Standard #6). Five microliters of each of these standards were added to microtubes containing 140 μL assay buffer and 5 μL of 10 mg/L beta-lactoglobulin. For the analytical samples, the standard solutions were substituted with human plasma or serum sample (diluted 2-fold into assay buffer).

### Mass spectrometric immunoassay

The antibody-derivatized affinity pipettes were mounted onto the head of the Multimek pipettor and initially rinsed with assay buffer (10 aspirations and dispense cycles, 100 μL volumes each). Next, the pipettes were immersed into a microplate containing the samples and 100 aspirations and dispense cycles were performed (100 μL volumes each) allowing for affinity capture of cystatin C and beta lactoglobulin. The pipettes were then rinsed with assay buffer (100 cycles), and twice with water (10 cycles each). In preparation of elution, the affinity pipettes containing the retrieved protein were rinsed with 1 mM N-octylglucoside (single cycle with a 150 μL aliquot) in order to homogenize the subsequent MALDI matrix draw and elution, by completely wetting the porous microcolumns inside the pipettes. For elution of the captured proteins, 6 μL aliquots of MALDI matrix (25 g/L α-cyano-4-hydroxycinnamic acid in aqueous solution containing 33% (v/v) acetonitrile, and 0.4% (v/v) trifluoroacetic acid) were aspired into the affinity pipettes, and after a 10 second delay (to allow for the dissociation of the protein from the capturing antibody), the eluates from all 96 affinity pipettes containing the targeted proteins were dispensed directly onto a 96-well formatted MALDI target. Following air-drying and visual inspection of the sample spots, linear mass spectra were acquired with a delayed extraction mode using a 1.7 kV draw out pulse, 200 ns delay, and a full accelerating potential of 20 kV. Five mass spectra were acquired from each sample spot, each spectrum consisting of three-hundred laser shots. The mass spectra were internally calibrated with the singly and doubly charged beta-lactoglobulin signals, and further processed (baseline subtracted and smoothed) with Flex Analysis software (Bruker Daltonics). The peak heights for the cystatin C and beta-lactoglobulin signals were measured and entered into an Excel spreadsheet. The ratios of the cysC/BL peak heights were calculated, and the average ratio for each sample determined. A standard curve was generated by plotting the CysC/BL ratios against the concentration of the human cystatin C standards, and the data was fitted with a linear trendline using Sigma Plot (Systat Software, San Jose, CA). This standard curve was then utilized to determine the absolute concentration of cystatin C and its variants in the analytical samples.

### Tryptic peptide mapping

Tryptic digestion was performed directly on the cystatin C eluates deposited onto the MALDI target after the mass spectrometric immunoassay of the human serum samples [6]. A 10 μL aliquot of 25 mM TRIS, pH 9.1, containing 0.5 mg/L trypsin (w/or w/o 1 mM DTT) was added onto each analytical sample spot and the entire MALDI plate was placed into a humidified enclave, at 40°C. To keep the samples solvated, one 10 μL aliquot of water was added to each spot at ~10 min into the digestion. Digestion was terminated after 20 min by air-drying the plate. The sample spots were re-hydrated with 5 μL aliquots of 0.8% TFA, and allowed to dry again. Following matrix re-crystallization, reflectron mass spectra were acquired with a delayed extraction mode using a 2.1 kV draw out pulse, 1100 ns delay, an ion mirror voltage of 20 kV, and a full accelerating potential of 19 kV

## Abbreviations

BL: beta-lactoglobulin; CysC: cystatin C; IRS: internal reference standard; MS: mass spectrometry.

## Competing interests

The authors declare that they have no competing interests.

## Authors' contributions

OT performed the experiments and analyzed the data. DN designed the experiments and drafted the manuscript. All authors read and approved the final manuscript.

## Authors' information

OT's permanent address is at the Institute of Chemistry, Faculty of Natural Sciences, Sts. Cyril and Methodius University, Skopje, Republic of Macedonia.
